# Crystal structure of (*Z*)-3-allyl-5-(4-methyl­benzyl­idene)-2-sulfanyl­idene-1,3-thia­zolidin-4-one

**DOI:** 10.1107/S2056989015020460

**Published:** 2015-11-04

**Authors:** Rahhal El Ajlaoui, El Mostapha Rakib, Mohammed Chigr, Mohamed Saadi, Lahcen El Ammari

**Affiliations:** a Laboratoire de Chimie Organique et Analytique, Université Sultan Moulay Slimane, Faculté des Sciences et Techniques, Béni-Mellal, BP 523, Morocco; b Laboratoire de Chimie du Solide Appliquée, Faculté des Sciences, Université Mohammed V, Avenue Ibn Battouta, BP 1014, Rabat, Morocco

**Keywords:** crystal structure, 1,3-thia­zolidin-4-one, biological activity, rhodanine-based mol­ecules

## Abstract

In the title compound, C_14_H_13_NOS_2_, the atoms of the allyl group are disordered over two sets of sites, with an occupancy ratio of 0.559 (10):0.441 (10). The rhodanine ring makes a dihedral angle of 5.51 (12)° with the mean plane through the *p*-tolyl group. There are no specific inter­molecular inter­actions in the crystal packing.

## Related literature   

For biological activities of rhodanine-based mol­ecules, see: Tomasić & Masic (2009 ▸); Jiang *et al.* (2011 ▸); Bulic *et al.* (2009 ▸); Sing *et al.* (2001 ▸); Grant *et al.* (2000 ▸); Orchard *et al.* (2004 ▸); Cutshall *et al.* (2005 ▸); Sortino *et al.* (2007 ▸); Kesel (2003 ▸).
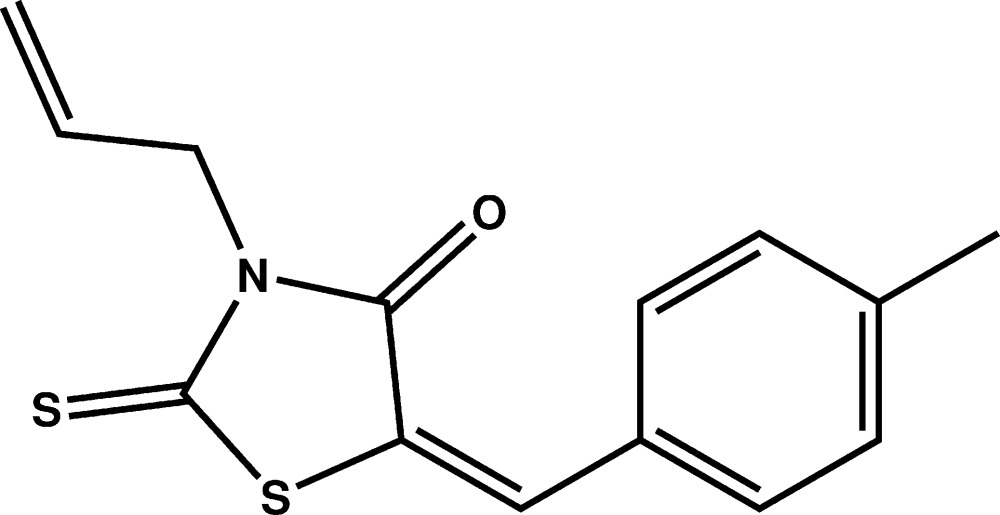


## Experimental   

### Crystal data  

C_14_H_13_NOS_2_
*M* *_r_* = 275.37
Triclinic, 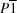

*a* = 7.3606 (4) Å
*b* = 8.8342 (6) Å
*c* = 11.3134 (7) Å
α = 109.736 (2)°
β = 95.380 (2)°
γ = 96.502 (2)°
*V* = 681.10 (7) Å^3^
*Z* = 2
Mo *K*α radiation
μ = 0.38 mm^−1^
*T* = 296 K
0.37 × 0.35 × 0.28 mm

### Data collection  

Bruker X8 APEX diffractometer
Absorption correction: multi-scan (*SADABS*; Bruker, 2009 ▸) *T*_min_ = 0.700, *T*_max_ = 0.746
21519 measured reflections
2853 independent reflections
2241 reflections with *I* > 2σ(*I*)
*R*_int_ = 0.032

### Refinement  

*R*[*F* ^2^ > 2σ(*F* ^2^)] = 0.043
*wR*(*F* ^2^) = 0.133
*S* = 1.07
2853 reflections
182 parameters
2 restraints
H-atom parameters constrained
Δρ_max_ = 0.34 e Å^−3^
Δρ_min_ = −0.21 e Å^−3^

### 

Data collection: *APEX2* (Bruker, 2009 ▸); cell refinement: *SAINT* (Bruker, 2009 ▸); data reduction: *SAINT*; program(s) used to solve structure: *SHELXS97* (Sheldrick, 2008 ▸); program(s) used to refine structure: *SHELXL97* (Sheldrick, 2008 ▸); molecular graphics: *ORTEPIII* (Burnett & Johnson, 1996 ▸) and *ORTEP-3 for Windows* (Farrugia, 2012 ▸); software used to prepare material for publication: *PLATON* (Spek, 2009 ▸) and *publCIF* (Westrip, 2010 ▸).

## Supplementary Material

Crystal structure: contains datablock(s) I. DOI: 10.1107/S2056989015020460/im2472sup1.cif

Structure factors: contains datablock(s) I. DOI: 10.1107/S2056989015020460/im2472Isup2.hkl

Supporting information file. DOI: 10.1107/S2056989015020460/im2472Isup3.cml

**Fig. 1.**
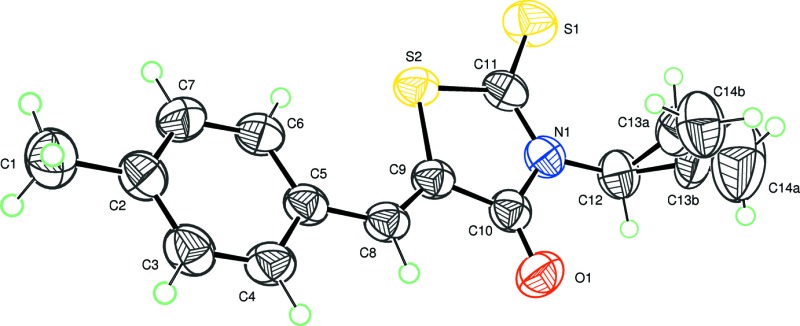
Plot of the molecule of the title compound with displacement ellipsoids are drawn at the 50% probability level. H atoms are represented as small circles.

. DOI: 10.1107/S2056989015020460/im2472fig1.tif
Plot of the mol­ecule of the title compound with displacement ellipsoids are drawn at the 50% probability level. H atoms are represented as small circles.

CCDC reference: 1433844

Additional supporting information:  crystallographic information; 3D view; checkCIF report

## supplementary crystallographic information

### S1. Structural commentary

Rhodanine-based molecules are known to possess diverse biological activities (Tomasic & Masic, 2009) through the inhibition of numerous targets such as HIV-1 (Jiang *et al.* 2011), Alzheimer's deseases (Bulic *et al.* 2009), HCV NS3 protease (Sing *et al.* 2001), β-la­cta­mase (Grant *et al.* 2000), PMT1 mannosyl transferase (Orchard *et al.* 2004), PRL-3 and JSP-1 phosphatases (Cutshall *et al.* 2005). Additionally, they have been reported to possess anti­microbial (Sortino *et al.* 2007) and anti­viral (Kesel, 2003) activities. The unusual biological activity displayed by many rhodanine-based molecules have made them attractive synthetic targets.

The rhodanine and p-tolyl ring systems (S2—N1—C9—C10—C11 and C2 to C7) are slightly inclined as indicated by the dihedral angle of 5.51 (12)° between them. The rhodanine moiety is linked to an allyl group at the nitro­gen atom and to a p-tolyl group at C(5) as shown in Fig.1. Moreover, the molecule of the title compound is characterized by a disorder in the allyl group in which all atoms are split with an occupancy factors of 0.559 (9) : 0.441 (9). No specific inter­molecular inter­actions are observed in the crystal packing.

### S2. Synthesis and crystallization

To a solution of 3-allyl­rhodanine (1.15 mmol, 0.2 g) in 10 ml of THF, (4-methyl­benzyl­idene)-4-methyl-5-oxopyrazolidin-2-ium-1-ide (1.38 mmol) was added. The mixture was refluxed for 8 h, monitored by TLC, the reaction completed and a yellow spot (TLC R_f_ = 0.3, using hexane/ethyl acetate 1:9) was generated cleanly. The solvent was evaporated in vacuo. The crude product was purified on silica gel using hexane: ethyl acetate (1/9) as eluent. The title compound was recrystallized from ethanol (Yield: 75%; m.p.: 390 K).

### S3. Refinement

Crystal data, data collection and structure refinement details are summarized in Table 1. The reflection (0 0 1) affected by the beamstop was removed during refinement. The refinement of the model requires constraints on the distance C13A—C14A and C13B—C14B of the disordered allyl group. H atoms were located in a difference map and treated as riding with C–H = 0.96 Å, C–H = 0.97 Å and C–H = 0.93 Å for methyl, methyl­ene and aromatic hydrogen atoms, respectively. All hydrogen atoms were refined with a common thermal displacement parameter of U_iso_(H) = 1.5 U_eq_ for methyl and U_iso_(H) = 1.2 U_eq_ for methyl­ene and aromatic hydrogen atoms.

### Crystal data

**Table:**

C_14_H_13_NOS_2_	*F*(000) = 288
*M**_r_* = 275.37	*D*_x_ = 1.343 Mg m^−^^3^
Triclinic, *P*1	Melting point: 390 K
*a* = 7.3606 (4) Å	Mo *K*α radiation, λ = 0.71073 Å
*b* = 8.8342 (6) Å	Cell parameters from 2853 reflections
*c* = 11.3134 (7) Å	θ = 2.5–26.6°
α = 109.736 (2)°	µ = 0.38 mm^−^^1^
β = 95.380 (2)°	*T* = 296 K
γ = 96.502 (2)°	Block, yellow
*V* = 681.10 (7) Å^3^	0.37 × 0.35 × 0.28 mm
*Z* = 2	

### Data collection

**Table:**

Bruker X8 APEX diffractometer	2853 independent reflections
Radiation source: fine-focus sealed tube	2241 reflections with *I* > 2σ(*I*)
Graphite monochromator	*R*_int_ = 0.032
φ and ω scans	θ_max_ = 26.6°, θ_min_ = 2.5°
Absorption correction: multi-scan (*SADABS*; Bruker, 2009)	*h* = −9→9
*T*_min_ = 0.700, *T*_max_ = 0.746	*k* = −11→11
21519 measured reflections	*l* = −14→14

### Refinement

**Table:**

Refinement on *F*^2^	2 restraints
Least-squares matrix: full	Hydrogen site location: inferred from neighbouring sites
*R*[*F*^2^ > 2σ(*F*^2^)] = 0.043	H-atom parameters constrained
*wR*(*F*^2^) = 0.133	*w* = 1/[σ^2^(*F*_o_^2^) + (0.0651*P*)^2^ + 0.2196*P*] where *P* = (*F*_o_^2^ + 2*F*_c_^2^)/3
*S* = 1.07	(Δ/σ)_max_ < 0.001
2853 reflections	Δρ_max_ = 0.34 e Å^−^^3^
182 parameters	Δρ_min_ = −0.21 e Å^−^^3^

### Special details

**Table:**

Geometry. All e.s.d.'s (except the e.s.d. in the dihedral angle between two l.s. planes) are estimated using the full covariance matrix. The cell e.s.d.'s are taken into account individually in the estimation of e.s.d.'s in distances, angles and torsion angles; correlations between e.s.d.'s in cell parameters are only used when they are defined by crystal symmetry. An approximate (isotropic) treatment of cell e.s.d.'s is used for estimating e.s.d.'s involving l.s. planes.

### Fractional atomic coordinates and isotropic or equivalent isotropic displacement parameters (Å^2^)

**Table:**

	*x*	*y*	*z*	*U*_iso_*/*U*_eq_	Occ. (<1)
C1	0.5130 (4)	0.6507 (3)	1.2642 (3)	0.0784 (7)	
H1A	0.3857	0.6024	1.2376	0.118*	
H1B	0.5440	0.6723	1.3533	0.118*	
H1C	0.5329	0.7509	1.2481	0.118*	
C2	0.6328 (3)	0.5359 (3)	1.1916 (2)	0.0591 (5)	
C3	0.8224 (3)	0.5758 (3)	1.2117 (2)	0.0644 (6)	
H3	0.8773	0.6754	1.2720	0.077*	
C4	0.9323 (3)	0.4717 (3)	1.1444 (2)	0.0608 (6)	
H4	1.0599	0.5021	1.1607	0.073*	
C5	0.8563 (3)	0.3212 (2)	1.05205 (18)	0.0507 (5)	
C6	0.6656 (3)	0.2794 (3)	1.0338 (2)	0.0604 (5)	
H6	0.6103	0.1791	0.9747	0.073*	
C7	0.5571 (3)	0.3847 (3)	1.1022 (2)	0.0659 (6)	
H7	0.4296	0.3536	1.0882	0.079*	
C8	0.9804 (3)	0.2217 (3)	0.98157 (19)	0.0530 (5)	
H8	1.1048	0.2647	1.0078	0.064*	
C9	0.9485 (3)	0.0782 (3)	0.88491 (19)	0.0518 (5)	
C10	1.1041 (3)	0.0018 (3)	0.8283 (2)	0.0552 (5)	
C11	0.8509 (3)	−0.1882 (3)	0.7007 (2)	0.0594 (5)	
C12	1.1670 (4)	−0.2379 (3)	0.6539 (2)	0.0695 (6)	
H12A	1.1258	−0.3537	0.6306	0.083*	
H12B	1.2900	−0.2102	0.7018	0.083*	
C13A	1.1640 (13)	−0.1898 (15)	0.5393 (11)	0.090 (3)	0.441 (10)
H13A	1.0526	−0.2181	0.4852	0.108*	0.441 (10)
C14A	1.3042 (16)	−0.1105 (14)	0.5064 (12)	0.147 (5)	0.441 (10)
H14A	1.4185	−0.0794	0.5573	0.177*	0.441 (10)
H14B	1.2876	−0.0863	0.4326	0.177*	0.441 (10)
C13B	1.2460 (12)	−0.1897 (10)	0.5554 (8)	0.077 (2)	0.559 (10)
H13B	1.3415	−0.2408	0.5196	0.092*	0.559 (10)
C14B	1.1877 (12)	−0.0760 (8)	0.5147 (6)	0.101 (3)	0.559 (10)
H14C	1.0923	−0.0231	0.5491	0.121*	0.559 (10)
H14D	1.2423	−0.0497	0.4519	0.121*	0.559 (10)
N1	1.0378 (3)	−0.1426 (2)	0.72653 (17)	0.0560 (4)	
O1	1.2661 (2)	0.0540 (2)	0.86169 (17)	0.0734 (5)	
S1	0.73489 (11)	−0.35069 (9)	0.59005 (7)	0.0874 (3)	
S2	0.73980 (7)	−0.04619 (7)	0.80585 (5)	0.0607 (2)	

### Atomic displacement parameters (Å^2^)

**Table:**

	*U*^11^	*U*^22^	*U*^33^	*U*^12^	*U*^13^	*U*^23^
C1	0.0840 (18)	0.0719 (16)	0.0752 (17)	0.0164 (14)	0.0191 (14)	0.0170 (14)
C2	0.0674 (13)	0.0592 (13)	0.0517 (12)	0.0074 (10)	0.0086 (10)	0.0217 (10)
C3	0.0730 (15)	0.0523 (12)	0.0566 (12)	−0.0057 (11)	0.0068 (11)	0.0102 (10)
C4	0.0523 (11)	0.0603 (13)	0.0602 (13)	−0.0082 (10)	0.0000 (10)	0.0165 (11)
C5	0.0541 (11)	0.0526 (11)	0.0445 (10)	−0.0004 (9)	0.0024 (8)	0.0200 (9)
C6	0.0567 (12)	0.0552 (12)	0.0564 (12)	−0.0027 (10)	−0.0022 (10)	0.0093 (10)
C7	0.0497 (12)	0.0697 (14)	0.0679 (14)	0.0011 (10)	0.0030 (10)	0.0149 (12)
C8	0.0503 (11)	0.0537 (11)	0.0523 (11)	−0.0027 (9)	0.0008 (9)	0.0201 (9)
C9	0.0527 (11)	0.0529 (11)	0.0502 (11)	0.0019 (9)	0.0022 (9)	0.0218 (9)
C10	0.0592 (12)	0.0566 (12)	0.0521 (11)	0.0056 (10)	0.0077 (9)	0.0231 (10)
C11	0.0710 (14)	0.0527 (12)	0.0531 (12)	0.0057 (10)	−0.0007 (10)	0.0204 (10)
C12	0.0810 (16)	0.0628 (14)	0.0660 (14)	0.0176 (12)	0.0197 (12)	0.0197 (12)
C13A	0.078 (6)	0.123 (8)	0.077 (5)	0.049 (7)	0.023 (5)	0.030 (5)
C14A	0.171 (11)	0.176 (11)	0.163 (10)	0.100 (10)	0.091 (9)	0.106 (9)
C13B	0.064 (4)	0.102 (4)	0.081 (4)	0.033 (4)	0.036 (4)	0.040 (3)
C14B	0.128 (7)	0.105 (5)	0.092 (4)	0.026 (4)	0.043 (4)	0.054 (4)
N1	0.0635 (11)	0.0541 (10)	0.0526 (10)	0.0100 (8)	0.0082 (8)	0.0211 (8)
O1	0.0546 (9)	0.0745 (11)	0.0810 (11)	0.0037 (8)	0.0101 (8)	0.0164 (9)
S1	0.0934 (5)	0.0627 (4)	0.0795 (5)	0.0007 (3)	−0.0076 (4)	−0.0002 (3)
S2	0.0540 (3)	0.0597 (4)	0.0580 (3)	0.0004 (2)	−0.0020 (2)	0.0126 (3)

### Geometric parameters (Å, º)

**Table:**

C1—C2	1.502 (3)	C10—O1	1.204 (3)
C1—H1A	0.9600	C10—N1	1.397 (3)
C1—H1B	0.9600	C11—N1	1.364 (3)
C1—H1C	0.9600	C11—S1	1.633 (2)
C2—C3	1.378 (3)	C11—S2	1.751 (2)
C2—C7	1.390 (3)	C12—C13B	1.465 (8)
C3—C4	1.374 (3)	C12—N1	1.466 (3)
C3—H3	0.9300	C12—C13A	1.493 (12)
C4—C5	1.399 (3)	C12—H12A	0.9700
C4—H4	0.9300	C12—H12B	0.9700
C5—C6	1.388 (3)	C13A—C14A	1.3337 (10)
C5—C8	1.445 (3)	C13A—H13A	0.9300
C6—C7	1.378 (3)	C14A—H14A	0.9300
C6—H6	0.9300	C14A—H14B	0.9300
C7—H7	0.9300	C13B—C14B	1.3331 (10)
C8—C9	1.344 (3)	C13B—H13B	0.9300
C8—H8	0.9300	C14B—H14C	0.9300
C9—C10	1.482 (3)	C14B—H14D	0.9300
C9—S2	1.750 (2)		
			
C2—C1—H1A	109.5	O1—C10—N1	123.1 (2)
C2—C1—H1B	109.5	O1—C10—C9	126.5 (2)
H1A—C1—H1B	109.5	N1—C10—C9	110.46 (18)
C2—C1—H1C	109.5	N1—C11—S1	127.64 (18)
H1A—C1—H1C	109.5	N1—C11—S2	110.78 (16)
H1B—C1—H1C	109.5	S1—C11—S2	121.58 (15)
C3—C2—C7	117.3 (2)	C13B—C12—N1	119.7 (3)
C3—C2—C1	121.3 (2)	N1—C12—C13A	103.2 (5)
C7—C2—C1	121.4 (2)	N1—C12—H12A	111.1
C4—C3—C2	121.4 (2)	C13A—C12—H12A	111.1
C4—C3—H3	119.3	N1—C12—H12B	111.1
C2—C3—H3	119.3	C13A—C12—H12B	111.1
C3—C4—C5	121.4 (2)	H12A—C12—H12B	109.1
C3—C4—H4	119.3	C14A—C13A—C12	126.8 (11)
C5—C4—H4	119.3	C14A—C13A—H13A	116.6
C6—C5—C4	117.1 (2)	C12—C13A—H13A	116.6
C6—C5—C8	124.73 (19)	C13A—C14A—H14A	120.0
C4—C5—C8	118.14 (19)	C13A—C14A—H14B	120.0
C7—C6—C5	120.8 (2)	H14A—C14A—H14B	120.0
C7—C6—H6	119.6	C14B—C13B—C12	123.2 (7)
C5—C6—H6	119.6	C14B—C13B—H13B	118.4
C6—C7—C2	121.8 (2)	C12—C13B—H13B	118.4
C6—C7—H7	119.1	C13B—C14B—H14C	120.0
C2—C7—H7	119.1	C13B—C14B—H14D	120.0
C9—C8—C5	131.6 (2)	H14C—C14B—H14D	120.0
C9—C8—H8	114.2	C11—N1—C10	116.69 (19)
C5—C8—H8	114.2	C11—N1—C12	123.1 (2)
C8—C9—C10	120.57 (19)	C10—N1—C12	120.2 (2)
C8—C9—S2	130.12 (17)	C9—S2—C11	92.73 (10)
C10—C9—S2	109.31 (15)		
