# Supplementary figures and images for: Crystal structure of (Z)-3-allyl-5-(4-methyl­benzyl­idene)-2-sulfanyl­idene-1,3-thia­zolidin-4-one
Source: Acta Crystallogr E Crystallogr Commun. 2015 Nov 4;71(Pt 12):o906–7. doi: 10.1107/S2056989015020460 (PMC4719873; doi:10.1107/S2056989015020460)

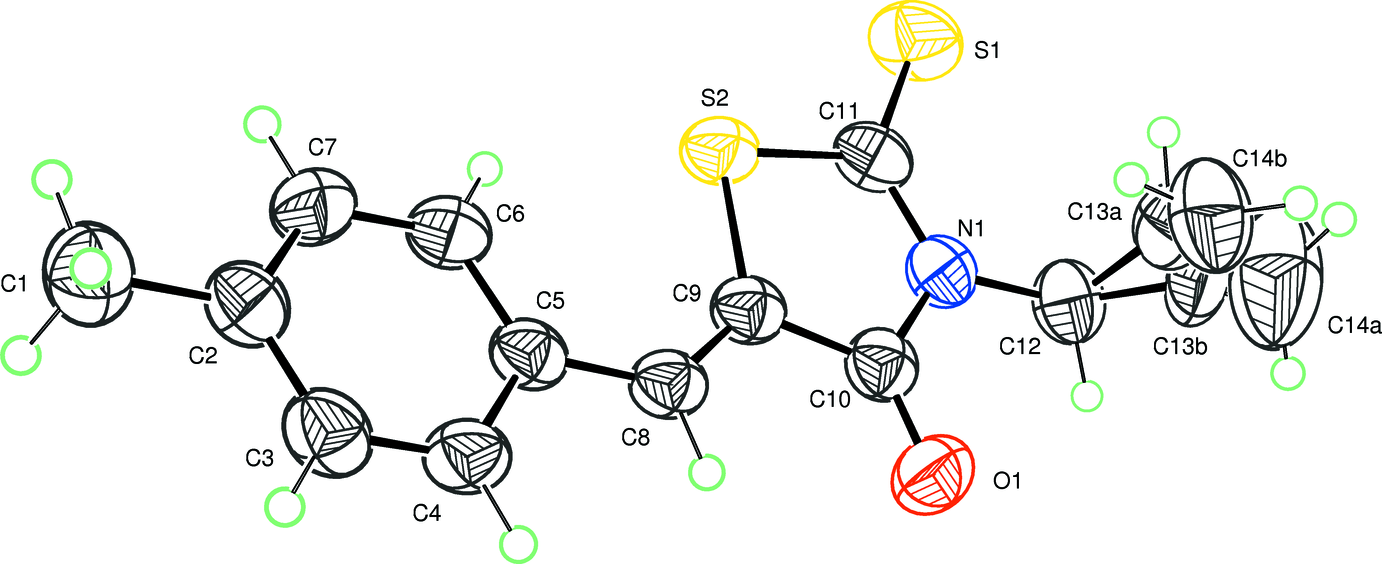

Supplement: Supplementary file 4 [file e-71-0o906-fig1.tif]
